# Problem-based learning for radiological technologists: a comparison of student attitudes toward plain radiography

**DOI:** 10.1186/s12909-016-0753-7

**Published:** 2016-09-05

**Authors:** Takayoshi Terashita, Naomi Tamura, Kengo Kisa, Hidenobu Kawabata, Katsuhiko Ogasawara

**Affiliations:** 1 Faculty of Health Sciences, Hokkaido University, Kitaku N12 W5, Sapporo, Hokkaido 060-0821 Japan; 2 Graduate School of Health Sciences, Hokkaido University, Kitaku N12 W5, Sapporo, Hokkaido 060-0821 Japan; 3 Kutchan-Kosei General Hospital, N4 E1, Kutchan, Hokkaido 044-0004 Japan; 4 Department of Medical Education and General Medicine, Graduate School of Medicine, Hokkaido University, Kitaku N15 W7, Sapporo, Hokkaido Japan

**Keywords:** Problem-based learning, Student’s attitude, Semantic differential technique, Factor analysis, Radiological technologist, Plain radiography practical training, Self-efficacy

## Abstract

**Background:**

Knowledge and skill expected of healthcare providers continues to increase alongside developments in medicine and healthcare. Problem-based learning (PBL) is therefore increasingly necessary in training courses for radiological technologists. However, it is necessary to evaluate the effects of PBL to completely introduce it in our education programs. As a Hypothesis, it seems that a change occurs in the student’s attitudes by participating in PBL practical training. There is the Semantic Differential (SeD) technique as a method to identify student’s attitudes. We conceived that PBL could be appropriately evaluated by using SeD technique. In this paper, we evaluated PBL for plain radiography practical training using the SeD technique.

**Methods:**

Thirty-eight third-year students studying radiological technology participated. PBL was introduced to practical training in plain radiography positioning techniques. Five sessions lasting 5 h each were delivered over a 5-week period during November to December 2012. The clinical scenario was an emergency case with multiple trauma requiring plain radiography. Groups comprising approximately eight students created workflows for trauma radiography with consideration of diagnostic accuracy and patient safety. Furthermore, students groups conducted plain radiography on a patient phantom according to created workflows and were then guided by feedback from professional radiologists. All students answered SeD questionnaires to assess views on plain radiography before instruction to provide preliminary practical training reports and after completing practical training.

**Results:**

The factors were identified using factor analysis of the questionnaires, which were answered before and after each practical training session. On evaluation of the relationships between factors and question items according to factor loading, we identified “reluctance”, “confidence”, and “exhaustion” as the predominant attitudes before practical training. Similarly, we identified “expectation”, “self-efficacy”, and “realness” as the predominant attitudes after practical training. The attitudes toward plain radiography changed before and after PBL practical training.

**Conclusions:**

The attitude of self-efficacy was noted after practical training, which incorporated PBL. Student self-efficacy was thought to increase through self-directed learning, which is one of the aims of PBL. Although the influences of other lectures and training, which were performed in parallel with the PBL practice training, were not completely excluded, and although the number of study participants was small, we were able to confirm the effects of PBL.

**Electronic supplementary material:**

The online version of this article (doi:10.1186/s12909-016-0753-7) contains supplementary material, which is available to authorized users.

## Background

The knowledge and skills expected of healthcare providers continue to increase alongside developments in medicine and healthcare. Healthcare providers must keep up to date with the latest evidence and techniques along with their substantial clinical duties. However, the teaching of new evidence and techniques and related clinical training is challenging. Rapid progress in research, inefficiency of cramming, and fact knowledge becomes obsolete after 10 years are all major issues in medical education. Therefore, training courses for doctors have recently focused on more efficient teaching methods for the development of knowledge and skills [1].

Similarly, training courses for healthcare workers need to change from conventional learning methods. For example in radiology, methods for viewing plain radiographs are changing from screen film systems to imaging plates, and flat panel detectors are increasingly being installed in general hospitals. Superconducting magnetic resonance imaging systems are changing from 0.5T to 1.5T, with 3.0T machines becoming more common. Radiological technologists need to possess the knowledge and skills that change over time including new imaging techniques and clinical image interpretation, information sharing, and healthcare safety resulting from developments in equipment [2]. Consequently, training that imparts all of this knowledge in a short time period will become increasingly challenging in the future, and training courses for healthcare professionals, including radiological technologists, will need to include more efficient learning methods.

One approach for achieving this aim is problem-based learning (PBL) [1]. PBL is a learning method involving the use of clinical scenarios to acquire knowledge which is then applied to improve problem-solving abilities. Advantages of PBL are that it allows students to develop independence as they learn according to their individual learning needs, that it stimulates reflection and self-directed learning for lifelong learning, cultivates critical thinking and evidence-based decision making, and that it supports effective teamwork and communication between colleagues. The practical use of PBL is already being widely adopted for medical education in Japan [3]. However, PBL is rarely included in training courses for radiological technologists in Japan and there have been few studies examining the role of PBL in this setting [4, 5]. Therefore, it is necessary to evaluate the effects of PBL to completely introduce it in our education programs.

On the other hand, a critical issue in education is the evaluation of training outcomes. Student portfolios and tests measuring achievement levels are generally used to evaluate PBL [1]. However, because knowledge and skills cover a diverse range of topics and PBL cultivates problem-solving abilities, methods used for evaluating training outcomes fail to address all of these areas. One method of evaluating training that has been used over a long period is the Semantic Differential (SeD) technique [6]. The SeD technique, developed in 1957 by Charles Osgood, is a technique for identifying attitudes [7]. The SeD technique is a questionnaire involving questions using bipolar adjective scales to measure participant impressions. Factor analysis of these data can be used to identify attitudes [8–10]. There are a number of previous studies suggesting that PBL can lead to improved student attitudes [11–14]. For example, Koponen et al. compared medical student attitudes in three experiential learning methods by using a communication skill attitude scale before and after each learning method [12]. Prince showed that adopting PBL positively influenced student attitude; however, the student test scores were not improved [11]. He also described difficulties in the analysis due to a variety of practices and the lack of a dominant core element associated with PBL. Although the exclusive attitude measurement scale is used if it is prepared, the types of attitudes for new learning methods should be examined widely. Khan et al. investigated medical students’ knowledge and attitudes for PBL and conventional curricula [13]. Attitudes toward health research, which was their theme, were investigated using original questionnaire items. Walton defined a cognitive element, an affective element, and a tendency to action as student attitude components [8]. Tendency to action among the three elements was the attitude intended for study. However, all attitudes were not covered in a questionnaire of researcher-fixed ideas. In a qualitative study, Sathishkumar et al. investigated medical student attitudes toward early clinical exposure [14]. Qualitative studies create a theory by generalizing various statements; however, quantity is often difficult to handle in a continuous investigation [15]. The SeD technique can be applied to common concepts and has the ability to perform continuous investigation for a quantitative study [6]. As a hypothesis, it seems that a change occurs in the student’s attitudes by participating in PBL practical training, for example because of stimulating the self-directed learning. The changes of attitude can identify by conducting SeD questionnaires before and after PBL practical training. We conceived that PBL could be appropriately evaluated by identifying the changes of attitude.

Hence, the purpose of this study is to introduce the PBL to radiological technology education, and to evaluate the effects of PBL by using the SeD technique. However, as testing for all curricula is challenging in this study, we need to narrow the focus. In this paper, we deal in the practical training for plain radiography positioning techniques.

## Methods

### Student participants and the practical application of PBL

We included 43 third-year students studying radiological technology at Hokkaido University. The PBL approach was introduced to practical training of positioning techniques for plain radiography. Students were divided into the small groups comprising approximately eight students. When the students were divided into groups, equal academic achievement in other lectures and training before PBL practice training was a consideration. A total five sessions lasting 5 h were delivered over a 5-week period during November and December 2012. Other specialized subjects, including ten lectures and four other types of training (eg, medical equipment, oncology, radiochemistry, radiation protection, etc.), were performed in parallel with the PBL practice training. The clinical scenario was an emergency case with multiple trauma requiring plain radiography (Appendix). Before this practical training experience, students were asked to research relevant knowledge required for this scenario. During practical training, students group discussed how to radiograph the lesions while considering diagnostic accuracy and patient safety. A plain radiograph workflow was created as the result of group work. Furthermore, students actually conducted plain radiography for a patient phantom according to the developed workflow, and then they were guided with professional’s feedback. After the practical training, students undertook the other clinical cases, considered appropriate radiographic methods and actions of radiological technologists. The institutional review board of the Hokkaido University Faculty of Health Sciences approved this study.

### Questionnaire survey using the SeD technique

We prepared a SeD questionnaire to evaluate the effects of PBL introduced into plain radiography practical training. Question items required adjectives relevant to plain radiography. Thus, we compiled sentences, including personal opinions of plain radiography, from reports of identical practical training sessions conducted from 2008 to 2010 [16]. We selected 50 adjectives from these sentences. Along with a further 50 adjectives with opposite meanings, we created 50 adjective pairs as question items. A visual analogue scale was used for the question format. The SeD questionnaire in this study was shown in Table 1. The impressions of all students regarding plain radiography were surveyed using the SeD questionnaire before being instructed to prepare preliminary reports for practical training (November 19, 2012) and after the entire group had completed practical training (January 17, 2013). In addition, after PBL practice training, a few students were asked about their impressions of the training. In this regard, asking and talking was not a systematic procedure as in a qualitative study.

**Table 1:**
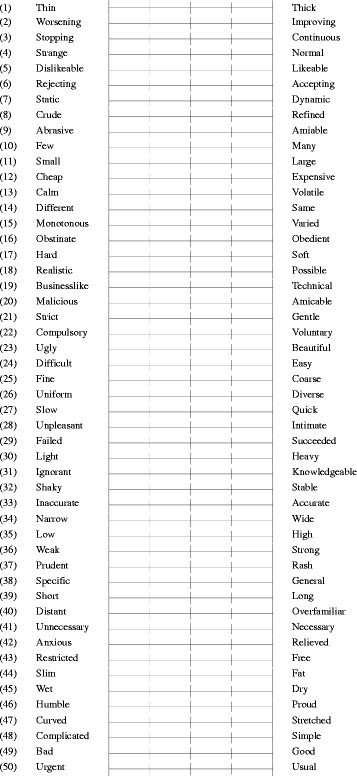
Semantic differential questionnaire in this study

### Analysis procedure

We performed factor analysis using adjective questions from the SeD questionnaire as dependent variables and compared attitudes identified in responses before and after practical training. Factors were estimated using maximum likelihood estimation and adopted varimax rotation. We further narrowed the number of variables suitable for analysis to improve the statistical power of factor analysis. In this study, the following criteria were used: an eigenvalue of 1 or more for identified factors, higher contribution ratio for factor analysis, and factor loadings of 0.5 or more. However, there were 50 questions in this study; because the number of possible combinations was essentially limitless, it was not possible to verify all of them. Therefore, using a genetic algorithm, we investigated the optimal variable combinations [17]. Genetic algorithms are used for optimization problems. We used the abovementioned criteria as evaluation functions. First, the expression of whether to set a variable for analysis as 0 or 1 was considered one gene and then 100 individuals were created randomly and assigned 0 or 1. Next, we performed factor analysis on each of these and selected 20 excellent individuals according to the abovementioned criteria. Next, 80 individuals were created from the selected excellent individuals by crossing and mutation. We again performed factor analysis using these 100 individuals and an operation to select excellent individuals. This process was repeated until 10,000 times. Finally, the best gene in the final generation was used as the optimal combination of variables for factor analysis. The Kruskal-Wallis test was performed using the factor score, which was calculated using the provided factors to confirm the deflection of each student group. We used R statistical software (version 2.15.0 R Foundation for Statistical Computing) for all analysis.

## Results

Table 2 shows attitudes toward plain radiography held by surveyed students before practical training. First, eight suitable items were selected for analysis: “Cheap/Expensive”, “Weak/Strong”, “Light/Heavy”, “Shaky/Stable”, “Inaccurate/Accurate”, “Anxious/Relieved”, “Short/Long”, and “Static/Dynamic.” Three factors were identified by eigenvalue analysis based on these items. The cumulative contribution ratio of these three factors was 61.6%. From the relationship between factors and adjectives based on factor loading, we interpreted Factor 1 as the attitude of “reluctance”, Factor 2 as “confidence”, and Factor 3 as “exhaustion.”

**Table 2:** Attitudes of students toward plain radiography before practical training (*n* = 43)

	Factor 1	Factor 2	Factor 3	Communality
Cheap/Expensive	0.897			0.821
Weak/Strong	0.743			0.560
Light/Heavy	0.714			0.564
Shaky/Stable		0.766		0.617
Inaccurate/Accurate		0.738		0.562
Anxious/Relieved		0.612		0.387
Short/Long			0.983	0.995
Static/Dynamic			0.624	0.418
Proportion (%)	24.0	19.1	18.5	61.6
Cumulative proportion (%)	24.0	43.1	61.6	
Factor name	Reluctance	Confidence	Exhaustion	

Table 3 shows attitudes toward plain radiography held by surveyed students following practical training. As in the previous procedure, 10 items were selected as suitable for analysis: “Rejecting/Accepting”, “Bad/Good”, “Unnecessary/Necessary”, “Low/High”, “Compulsory/Voluntary”, “Restricted/Free”, “Dry/Wet”, “Light/Heavy”, “Businesslike/Technical”, and “Complicated/Simple.” Eigenvalue analysis was used to identify three factors. The cumulative contribution ratio of the three identified factors was 63.4%. From the relationship between factors and adjectives based on factor loading, we interpreted Factor 1 as the attitude of “expectation”, Factor 2 as “self-efficacy”, and Factor 3 as “realness.” However, the impression given by English words for adjectives and factors may differ because this investigation was performed in Japanese. To minimize this, an English native speaker provided the most accurate translations possible.

**Table 3:** Attitudes of students toward plain radiography after practical training (*n* = 43)

	Factor 1	Factor 2	Factor 3	Communality
Rejecting/Accepting	0.922			0.868
Bad/Good	0.897			0.823
Unnecessary/Necessary	0.697			0.506
Low/High		0.859		0.794
Compulsory/Voluntary		0.749		0.619
Restricted/Free		0.618		0.605
Dry/Wet		0.545		0.369
Light/Heavy			0.906	0.823
Businesslike/Technical			0.671	0.471
Simple/Complicated			0.637	0.463
Proportion (%)	24.1	20.3	19.0	63.4
Cumulative proportion (%)	24.1	44.4	63.4	
Factor name	Expectation	Self-efficacy	Realness	

The factor score median and interquartile range for every group and the Kruskal-Wallis test results are shown in Table 4. There was no significant difference between the student groups for all factors (*P* value > 0.05). Therefore, the observed attitudes were considered to be the general effects of PBL practice participation. The raw data for all analysis was provided in Additional file 1.

**Table 4:** The factor score median and interquartile range for every group and the Kruskal-Wallis test results

	Factor	Group						P-value
		1	2	3	4	5	6	
Before	Factor1	0.877(1.43)	−0.081(1.01)	−0.087(1.60)	−0.158(0.49)	−0.778(0.51)	−0.818(1.37)	0.61
	Factor2	0.574(0.99)	−0.616(1.08)	0.155(1.18)	0.065(0.36)	0.318(1.25)	0.615(1.67)	0.20
	Factor3	0.468(1.44)	−0.476(0.95)	−0.637(0.92)	−0.493(0.71)	0.348(0.91)	0.016(1.44)	0.55
After	Factor1	−0.788(0.95)	0.489(1.14)	−0.278(1.28)	0.272(1.96)	0.426(0.94)	0.383(0.38)	0.44
	Factor2	−0.462(0.85)	−0.224(0.75)	0.289(1.23)	0.457(0.91)	0.316(2.17)	−0.238(0.65)	0.62
	Factor3	0.188(1.24)	−0.211(0.69)	0.036(0.74)	1.014(0.70)	0.180(0.72)	−0.231(1.00)	0.26

Median (interquartile range)

## Discussion

### Explanation of the attitudes

According to factor analysis results, we identified “reluctance”, “confidence”, and “exhaustion” as attitudes toward plain radiography before practical training, and “expectation”, “self-efficacy”, and “realness” as attitudes after practical training. There is presently no study that addresses evaluating effects of PBL using SeD technique, although we picked up the references widely. In this section, we explain the identified attitudes in this study.

First, in the results of before practical training, the attitude of “reluctance” represented whether students are feeling pressure toward the plain radiography. Generally, in plain radiography, patients are exposed to radiation and its failure cannot be tolerated. In addition, it is necessary to ask patients to cooperate during an X-ray examination and we believe that these factors add to the pressure felt by the students. It is believed that students had the impressions of “Expensive”, “Strong”, and “Heavy” because they felt pressured by plain radiography. The attitude of “confidence” reflected whether the students have confidence to conduct plain radiography. The plain radiography is a basic skill that the radiological technologists should have knowledge regarding [2]. Plain radiography has a long history, beginning with discovery of X-ray by Roentgen, and it has been clinically used since; however, its applicability has reduced because of emerging efficient modalities. The students learnt about these attributes of radiography from a previous lecture. Students were considered to have the impressions of “Stable”, “Accurate”, and “Relieved” because they were affected by the attributes of radiography. The attitude of “exhaustion” represented whether students have anxiety for radiography techniques used by radiological technologist. The radiography techniques generally involve moving a body. The students who have not experienced the actual medical scene may have believed that the plain radiography involves hard and exhausting work. It is believed that students had the impressions of “Long” and “Dynamic” because they thought that radiological technicians worked long hours and were very active.

Next, in the results of after practical training, the attitude of “expectation” indicated whether the students could recognize the role and future of plain radiography. An emergency clinical scenario was used in practical training incorporating PBL. This scenario emphasized on the advantages of plain radiography and helped the students realize the practical application of the plain radiography. Students might have considered radiography to be archaic compared to newer technology, such as CT, MRI. Although negative attitudes of “reluctance” and “exhaustion” appeared before the PBL training, the students changed their impressions to “Accepting”, “Good”, and “Necessary” after PBL practical training. Furthermore, the attitude of “realness” reflected whether the students experienced virtual X-ray examination in as more practical and applicable. In the PBL practical training, clinical scenarios that conformed to real clinical settings were used, and the students actually conducted plain radiography according to a developed workflow. Students had the impressions of “Heavy”, “Technical”, and “Complicated” through clinical experience rather than through textbooks. Finally, the attitude of “self-efficacy” reflected the effects of PBL. High motivation was indicated by the adjectives “High” and “Wet.” In Japanese, “Dry” means “no surge of emotions” and “Wet” means “a surge of emotions.” PBL is used to provide context and motivation for subsequent learning [11] and efficiently helps to elicit self-initiative and to obtain knowledge and skills [1]. Students were considered to have the impressions of “Voluntary” and “Free” because of the effects of PBL. Therefore, the identification of the attitude of self-efficacy was based on those adjectives.

### Effects of PBL in practical training

The curriculum at this school broadly comprises general courses in the first year, basic specialized courses in the second year, specialized applied courses in the third year, and practical clinical training in the fourth year. Before the introduction of practical training involving PBL, no lecture had used PBL, and there were very few lecture addressing clinical scenarios before the fourth year. When we heard the impressions of the practical training from students, they indicated that they had not previously had similar practical training and were positive regarding applying the knowledge and that this process had been helpful. By using their own knowledge and devising workflows in groups, students could obtain specific knowledge from practical training including important aspects and thought processes in clinical practice. In addition, by performing plain radiography according to workflow they themselves had devised, students could relate their knowledge to the work of radiological technologists. While perceptions of plain radiography were responsible for these attitudes before practical training, attitudes held after practical training changed to ones of based on practical experience of plain radiography. In addition, students reported increased self-efficacy following practical training. Of note, the attitude of self-efficacy was provided after practical training incorporating PBL. Self-efficacy is a theory of behavioral change as advocated by Bandura and is the belief that one is capable of making behavioral changes to obtain results [18]. Bandura reported that self-directed mastery experiences are arranged to reinforce a sense of personal efficacy. Prince stated that PBL typically involves significant amounts of self-directed learning on the part of the students [11]. Hence, an attitude of self-efficacy was observed using the SeD technique because student self-efficacy increased through the self-directed learning of PBL practical training. Reported increases in self-efficacy following practical training indicate the effect of PBL. We demonstrated the effectiveness of incorporating PBL into plain radiography practical training.

### Limitation of this study

Compliance bias is likely in educational study. Because the investigators are the teaching staff and study participants are the students. Therefore, it is possible that the students may have perceived that by giving teaching staff favorable responses they could improve their grades. As several items were included in this questionnaire and students only indicated the degree to which each adjective was true, it would not have been possible for students to determine whether a response was favorable or unfavorable. Thus, the study results are likely to be reliable. However, this change in attitude following practical training may have been due to attending lectures or other practical training during this teaching period. Students were not in an isolated environment during this study and approximately 1 month elapsed between the start and end of training. In this study, it is not possible to exclude the influence of other lectures, practical training, or self-instruction during this time period. In addition, as the number of participants was small, it was not possible to determine the statistical significance of these results. Future surveys are required that possibly include the creation of individual evaluation indices.

## Conclusion

In this study, we provided practical training in plain radiography that included a PBL approach and conducted a survey of the changes in attitudes toward plain radiography held by students before and after practical training using the SeD technique. The attitudes of “reluctance”, “confidence”, and “exhaustion” were identified before PBL practical training, and the attitudes of “expectation”, “self-efficacy”, and “realness” were identified after training. In addition, it is important that the attitude of self-efficacy appeared after PBL practical training. Although the influence of other lectures and types of training, which were in parallel with PBL practice training, were not completely excluded, and although the number of study participants was small, we were able to confirm the effects of PBL.

## Appendix

Clinical scenario

Patient information: 24-year-old male, with a traffic injury from hitting a guardrail when speeding above the legal limit in a passenger vehicle and failing to round the curve. He was transported by ambulance and had severe injuries around the head and face, with a Glasgow coma scale score of 8. He lay to a stretcher now in an X-ray examination room.

X-ray order: the skull (anterior-posterior and lateral view), Waters’ view, the cervical vertebra (anterior-posterior and lateral view), supine chest, and supine abdomen. However, because students explored only plain radiography during practical training in this study, this was the only test modality considered.

## Additional file

Additional file 1: Visual analog scale scores before and after practical training: This is raw data necessary for analysis in this study. Cross tables are composed of 43 students and 50 adjective pairs. Scores take values from 0.0 to 100.0. (XLSX 39 kb)

## Abbreviations

PBL: Problem-based learning
SeD: Semantic Differential
